# Electrospun Food Polysaccharides Loaded with Bioactive Compounds: Fabrication, Release, and Applications

**DOI:** 10.3390/polym15102318

**Published:** 2023-05-16

**Authors:** Zhenyu Lin, Hao Chen, Shengmei Li, Xiaolu Li, Jie Wang, Shanshan Xu

**Affiliations:** 1 Institute for Advanced Study, Shenzhen University, Shenzhen 518060, China; marcus.lzy@szu.edu.cn (Z.L.); mason.hao.chan@gmail.com (H.C.); seongmee88@outlook.com (S.L.); lixiaolu2020@email.szu.edu.cn (X.L.); jiewang1001@outlook.com (J.W.); 2 Key Laboratory of Optoelectronic Devices and Systems of Ministry of Education and Guangdong Province, College of Physics and Optoelectronic Engineering, Shenzhen University, Shenzhen 518060, China

**Keywords:** electrospun polysaccharide, bioactive compounds, controlled release, biodegradable polymers

## Abstract

Food polysaccharides are well acclaimed in the field of delivery systems due to their natural safety, biocompatibility with the human body, and capability of incorporating/releasing various bioactive compounds. Electrospinning, a straightforward atomization technique that has been attracting researchers worldwide, is also versatile for coupling food polysaccharides and bioactive compounds. In this review, several popular food polysaccharides including starch, cyclodextrin, chitosan, alginate, and hyaluronic acid are selected to discuss their basic characteristics, electrospinning conditions, bioactive compound release characteristics, and more. Data revealed that the selected polysaccharides are capable of releasing bioactive compounds from as rapidly as 5 s to as prolonged as 15 days. In addition, a series of frequently studied physical/chemical/biomedical applications utilizing electrospun food polysaccharides with bioactive compounds are also selected and discussed. These promising applications include but are not limited to active packaging with 4-log reduction against *E. coli*, *L. innocua,* and *S. aureus*; removal of 95% of particulate matter (PM) 2.5 and volatile organic compounds (VOCs); heavy metal ion removal; increasing enzyme heat/pH stability; wound healing acceleration and enhanced blood coagulation, etc. The broad potentials of electrospun food polysaccharides loaded with bioactive compounds are demonstrated in this review.

## 1. Introduction

Bioactive compounds have been gaining increasing interest due to their health benefits in multiple ways such as anti-oxidation [1,2], cancer prevention [3,4], obesity control [5,6], etc. Nevertheless, many bioactive compounds are susceptible to environmental impacts such as temperature, ultraviolet (UV) radiation, and oxidative degradation, with fairly low bioavailability [7,8,9,10]. In order to overcome these limitations, effective delivery systems should be developed to properly encapsulate bioactive compounds to expand their shelf lives and enhance their absorption. Encapsulation strategies for bioactive compounds have been extensively researched. From an industry viewpoint, the routine forms of encapsulation for bioactive compounds are mostly tablets or capsules. These solutions are highly developed and have been applied to drugs for many years, with demonstrated cost effectiveness [11,12,13,14]. Tablets or capsules effectively prevent the loaded substances from being exposed to oxygen and moisture, thus protecting the vulnerable bioactive compounds. However, they mostly do not help to increase bioavailability, which is key to receiving the expected health benefits. Nano-emulsion is a popular strategy for bioactive compound encapsulation and delivery. It is able to incorporate poorly bioavailable bioactive compounds into an amphiphilic system that is more compatible with the digestive environment, thus enhancing their bioavailability [15,16]. At the same time, nano-emulsion is able to entrap bioactive compounds within the dispersed phase that is surrounded by the continuous phase, protecting vulnerable bioactive compounds. However, during emulsion preparation, it is inevitable to input high shear, high pressure, and heat to homogenize the system and dissolve bioactive compounds in the oil phase, which causes high energy consumption, especially for upscale production, and also heat degradation to some sensitive bioactive compounds.

**Figure 1:**
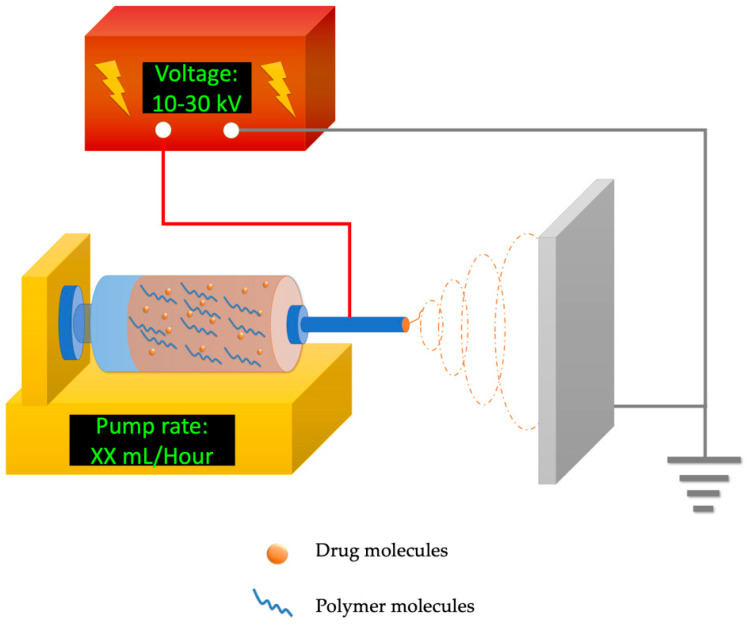
Typical electrospinning set–up to fabricate bioactive compound–loaded nanofibers, including a power source, an automatic syringe pump loaded with a polymer blend, and a grounded metal collector.

Electrospinning is a technique capable of encapsulating drugs within polymers in one step, which has attracted broad interest as an effective and efficient bioactive compound delivery system [17]. The set-up of electrospinning equipment is simple and includes a high-voltage power source (around 10–30 kilovolts (kV), an automatic syringe pump, and a grounded metal collector (demonstrated in Figure 1). The high-voltage electric field provides electrostatic force to the polymer blend at the needle tip, stretching it into ultrafine fibers and evaporating the solvents at the same time. Nanofibrous products are collected by the metal collector and accumulate into fibrous membranes [18]. Electrospinning techniques are helpful to enhance the bioavailability of many water-insoluble bioactive compounds [19] because of their high surface-to-volume ratio offered by the massive amount of nano-to-micron scale fibers inside. Some of the electrospun fibers possess porous surface structures that could further enhance the drug-carrying/releasing capability [20]. Electrospun materials are highly tunable by the flexible design of polymer formulations including polymer polarity, molecular weight, degradation duration, and more. In addition, through an adjustment of electrospinning techniques, special nanofiber types such as core–sheath, aligned, cross-fabrication, and layer by layer could also be created to satisfy various needs of drug-release profiles tailored for the delivery of bioactive compounds [21]. The active compound loading capacity of electrospun products could be much higher than routine emulsions or hydrogels [19,22]. In addition, electrospun products are mostly dry and thus are natively less susceptible to microbial impact due to low water activity. Therefore, electrospun products are more stable for storage and transport compared with other liquid or semi-liquid types of delivery systems. The multiple advantages of electrospinning demonstrate that it is an appealing solution to the challenges of bioactive compound encapsulation and delivery.

Food polysaccharides such as starch, cellulose, chitosan, alginate, and more, could be the most commonly seen food-based polymers in our daily lives. They are generally safe for humans to consume and highly biocompatible with human tissue [23]. Food polysaccharides exist abundantly in many types of food crops, edible algae, shellfish, and more, indicating their cost effectiveness in varied applications [24]. With these advantages, food polysaccharides have attracted researchers to turn them into drug delivery vehicles, including for bioactive compounds. Many food polysaccharides are able to be electrospun into nanofibers and encapsulate bioactive compounds at the same time, which could serve as an oral delivery system to enhance the bioavailability of bioactive compounds [25]. For some food polysaccharides that are difficult to electrospin (such as cellulose), modifications are popular solutions to enhance their ability for electrospinning. However, some modified food polysaccharides might fall outside of the U.S. Food and Drug Administration (FDA) approved categories and are not suitable for in vivo delivery of bioactive compounds.

In recent years, reviews on electrospun polysaccharides have concentrated very much on wound-healing- and tissue-engineering-related biomedical fields [26,27,28]. It was difficult to find comprehensive articles focusing on the release properties of electrospinning polysaccharides encapsulating bioactive compounds, which is actually important for the design and evaluation of their therapeutic efficacies. This review focuses mainly on electrospun food-based polysaccharides as carriers for bioactive compounds intended for in vivo delivery, and discusses their release characteristics by reviewing case studies. Applications other than the release of bioactive compounds, such as active packaging, filtering, enzyme immobilization, and wound dressing, will also be covered.

## 2. Food Polysaccharides for Electrospinning with Varied Release Characteristics

### 2.1. Starch

Starch is one of the most abundant polysaccharides that is available all over the world. It serves as an energy source for human beings and many living organisms; it is the safest type of polysaccharide with a low occurrence of allergic reactions (allergy is usually caused by other plant contents mixed with the starch, such as gluten). These appealing properties have attracted researchers to develop drug-delivery systems with starch. Nevertheless, due to the weak physical properties and the risk of creating drug–starch interactions, researchers tend to extensively modify starches by acetylation, oxidation, etc. These modified starch products might be unsafe for oral consumption or not approved for in vivo applications. It is challenging to develop delivery systems based on edible starch; however, a few successful examples of electrospun starch are reviewed here.

**Figure 2:**
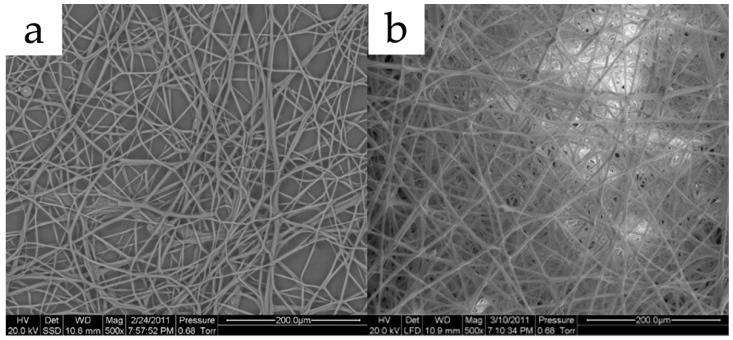
(**a**) Electrospun pure starch fibers under SEM; (**b**) electrospun starch–palmitic acid fibers. Reused with copyright permission from [29,36].

Kong and Ziegler reported that an 80% amylose corn starch was successfully electrospun into micron-scale fibers. During the electrospinning process, the solvent system was a 95% dimethyl sulfoxide (DMSO) aqueous solution in a boiling water bath for an hour. The metal collector was soaked in ethanol to help with fiber collection and washing of DMSO. The electric field was set at 7.5 kV over a 7.5 cm distance at room temperature while the polymer blend was fed at 4 mL/h. From their scanning electron microscope (SEM) images (demonstrated in Figure 2a), the starch fibers had an average diameter of around 2.6 microns with relatively normal distributions [29]. Although bioactive compound encapsulation was not arranged in their research, it was quite doable with their solvent system design. In another study by Li et al., a novel spinning method utilizing centrifugation forces was proven effective in the preparation of pure starch nanofibers. This technique evaporated starch solvents by centrifugation forces instead of electrostatic forces, therefore allowing for fewer requirements on the amylopectin content in starch and more flexible choices of solvent systems. The spinning set-up was self-prepared with needles of an inner diameter of 0.26 mm as nozzles, bent at 45°. The device completed 3000 revolutions per minute (rpm) and the distance to the collector was 6 cm. Hot air was utilized to speed up the solvent removal. They found that 2% (*w*/*w*) sodium hydroxide (NaOH) was suitable for successful centrifugal spinning for several types of common corn starch and even amylopectin-rich types at concentrations of around 8 to 16% [30].

Plenty of studies have researched the encapsulation of bioactive compounds in electrospun starch nanofibers, however, a large number of them tend to extensively modify the starch. Some studies utilized concentrated formic acid solution to dissolve edible starch, leading to intensive acetylation of starch [31], which is thus unsuitable for oral delivery. Some other researchers utilized starch to blend with non-digestible polymers to encapsulate bioactive compounds, which are also not intended for oral consumption [32,33,34,35]. Difficulties with electrospinning pure starch led to few achievements in the development of electrospun starch as a delivery system for bioactive compounds. One example was an inclusion form of starch-bioactive compound complex fibers successfully fabricated through electrospinning by Kong and Ziegler. They applied a 95% DMSO aqueous solution in a boiling water bath as their solvent system to untangle starch molecule clusters and thoroughly mix them with bioactive compounds. The electrospinning condition was set at 7.5 kV over 7.5 cm while the feeding rate was 4 mL/h at room temperature. After electrospinning, the high amylose starch formed an inclusion complex with palmitic acid (SEM image demonstrated in Figure 2b) [36]. Brief comparisons with other polysaccharides are listed in Table 1. Further data on the release of bioactive compounds from electrospun edible starches have been rarely seen; hopefully, more efforts will be invested into the development of an electrospun edible starch in the future.

### 2.2. Cyclodextrins

**Figure 3:**
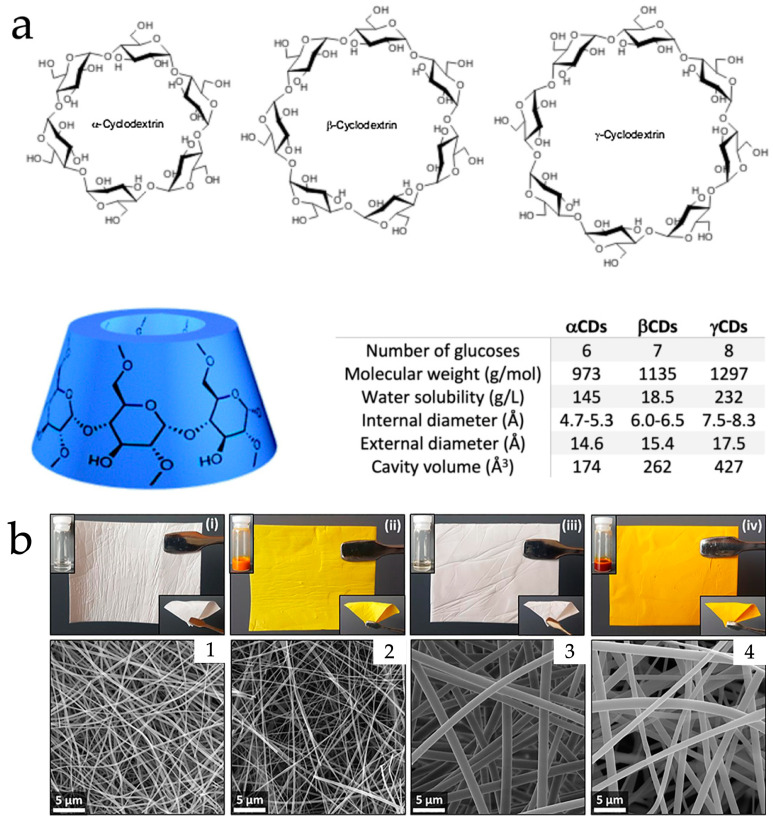
(**a**) Molecular structure and information of α, β, and γ-CD; (**b**) product appearances and corresponding SEM images of electrospun (**i**,**1**) HP-β-CD, (**ii**,**2**) curcumin/HP-β-CD, (**iii**,**3**) HP-γ-CD, and (**iv**,**4**) curcumin/HP-γ-CD. Reused with copyright permission from [38,57].

Cyclodextrins (CD) are a series of α (1 → 4)-linked cyclic glucopyranose. Natural CDs with six, seven, or eight glucose units are called α, β, or γ-CD [37]. Their spacial structures consist of characteristic funnel-like hollowed truncated cones, of which the interior cavity surface consists of relatively hydrophobic hydrocarbon backbones while the exterior is hydrophilic due to the protruding hydroxyl groups (demonstrated in Figure 3a) [37,38]. CD structures allow the encapsulation of hydrophobic drugs and bioactive compounds within the cavity to enhance their solubility and bioavailability [39,40,41,42]. Moreover, the safety of CDs is ensured by an adequate number of studies and official approvals. The Japanese Pharmaceutical Codex approved α, β, and γ-CD as food additives [43], while the FDA approved β-CD as GRAS in food [44]. The FDA also approved several products containing modified β-CD including sulfobutylether(SBE)-β-CD and hydroxypropyl(HP)-β-CD [45].

The utilization of CD in electrospinning can be performed in two ways. One way is to load the bioactive compound–CD inclusion complex into the electrospun polymer matrix. For this type of system, the fabrication process could be as straightforward as blending the CD–bioactive compound complex and the polymeric materials altogether and electrospinning them in one step. However, this direct-blending technique requires a solvent system that is compatible with both the polymer and the CD complex [46,47,48]. For incompatible situations, the core–sheath electrospinning technique could be helpful. It was achieved by preparing the CD–bioactive compound complex formulation as the core, and the polymer matrix as the shell, with the help of a special core–shell needle tip and a dual-syringe set-up [49,50,51]. The electrospun polymer matrix was able to protect the CD–bioactive compound complex to the extent that it could significantly increase the shelf-life and resistance to environmental impacts such as temperature and UV light [52]. The electrospun polymer matrix could also act as a reservoir to release the incorporated CD-bioactive compound in a gradual pattern, creating a prolonged/controlled-release profile of bioactive compounds. The other way to utilize CDs in electrospinning is to fabricate a polymer-free electrospun CD, which means the electrospun product matrix contains only a CD without other supporting polymers. In this technique, the CD actually self-assembled into an entangling and overlapping engagement with the polymer-like chain structure under the process of electrospinning through supramolecular interactions [53]. The polymer-free electrospun CD tends to perform a fast and enhanced release of the bioactive compounds or drugs loaded inside.

For the electrospun processes of the CD inclusion complex type of nanofibers, Hu et al. fabricated an electrospun polyvinyl alcohol (PVA) nanofibers encapsulating a berberine-HP-β-CD inclusion complex, using water as the solvent system with a PVA concentration of 8% and berberine at 0.5 to 1.5% *w*/*v*. The electric field was adjusted to 13 kV over a 10 cm distance with a feeding rate of 1 mL/h [46]. Aytac et al. electrospun a zein-encapsulated freeze-dried quercetin-γ-CD complex. A 35% (*w*/*v*) zein solution was prepared in 80% ethanol aqueous solution, while the quercetin-γ-CD complex was added at 5% *w*/*w* of zein. An electric field was applied at 15 kV over 10 cm with a feeding rate of 1 mL/h [54]. Celebioglu and Uyar developed an electrospun pullulan loaded with an eugenol-γ-CD complex. They prepared eugenol-γ-CD in water and pullulan (20%, *w*/*v*) was subsequently added to form the polymer blend. The voltage was set at 15 kV over 15 cm while the feeding rate was 0.5 mL/h using a 27-gauge (G) needle tip [55]. For the polymer-free type of electrospun CD, Sharif et al. incorporated an antibacterial bioactive compound cuminaldehyde into HP-β-CD to be electrospun into polymer-free CD nanofibers using water as a solvent system. The electric field was 15 kV over a 13 cm distance at ambient conditions while the feeding rate was 1 mL/h (needle inner diameter of 0.9 mm) [56]. Celebioglu et al. successfully enhanced the solubility of the water-insoluble bioactive compound curcumin via electrospun polymer-free cyclodextrin. They prepared a curcumin–CD inclusion complex by mixing curcumin suspension and CDs in 60 °C hot water for 24 h. The electrospinning voltage was 15 kV over a distance of 15 cm at ambient conditions. The feeding rate was 0.5 mL/h using a 23 G metal needle [57]. Song et al. fabricated polymer-free electrospun gallic acid/2-HP-β-CD inclusion complex nanofibers to overcome the limited aqueous solubility of gallic acid as a phenolic compound. An inclusion complex was prepared by 24 h stirring of gallic acid powder in an HP-β-CD solution. The solution was then electrospun to produce polymer-free HP-β-CD gallic acid nanofibers, with an electric field at 17 kV over 20 cm and a feeding rate of 0.7 mL/h using a metal needle of 18 G [48].

From the in vitro release results, Hu et al. reported that the electrospun PVA-berberine-HP-β-CD was able to achieve prolonged release of berberine for around four hours, while the raw berberine and electrospun PVA–berberine (without CD) could only provide a burst release to peak concentration in less than one hour. From their antibacterial effect results (*E. coli* and *S. aureus*), the CD-complexed berberine displayed a larger bacteria-free zone compared with the samples without CD complexation [46]. Celebioglu and Uyar demonstrated the release of eugenol from electrospun pullulan loaded with the eugenol-γ-CD complex was apparently enhanced from ~40 μg/mL (without CD) to ~160 μg/mL (with CD) in 2 min [55]. Sharif et al. found the electrospun HP-β-CD fibers able to release cuminaldehyde rapidly in an aqueous environment within five seconds while the raw powdery cuminaldehyde was unable to dissolve. Antibacterial effect against *E. coli* and *S. aureus* was observed for both cuminaldehyde samples in raw powder form and electrospun HP-β-CD fibers [56]. Celebioglu et al. achieved a clear curcumin solution rapidly by releasing the electrospun curcumin-HP-γ-CD. For the HP-β-CD complex, a slightly cloudy solution appeared (curcumin-loaded nanofibers and related SEM images are provided in Figure 3b) [57]. Song et al. prepared electrospun gallic acid–CD and it disintegrated in water within 5 s and achieved apparently higher gallic acid concentration compared with raw powder evaluated by UV spectroscopy. In a diphenyl picryl hydrazinyl (DPPH) radical scavenging assay for electrospun samples in a concentration under 100 μg/mL, the antioxidation activity appeared more than 1× stronger than powdery gallic acid [48]. Brief comparisons with other polysaccharides are listed in Table 1. Electrospun formulations and release profiles are summarized in Table 2.

### 2.3. Chitosan

Chitosan is a polysaccharide comprised of β-(1–4) glycosidic-linked glucosamine and N-acetylglucosamine [58]. It is generated by alkaline deacetylation of chitin (a natural polymer abundantly seen in crustaceans and insects) with a degree of deacetylation of over 50% [59]. The molecular weight (Mw) of chitosan on the market is widely distributed between 3800 and 2,000,000 [60]. The rectal and colonic enzymatic degradation speed of chitosan is negatively related to its degree of deacetylation and also the Mw [61]. Chitosan possesses multiple advantages that have attracted a large number of researchers in developing wound dressings, drug delivery systems, and more. First of all, chitosan is derived from the deacetylation of chitin, which generates a large number of amino groups that are able to be protonated [62]. This positively charged feature of chitosan contributes to its anti-bacterial properties since it has a charge affinity to the bacterial cell wall that is negatively charged (due to the ionized phosphoryl and carboxylate substituents on the bacterial cell wall [63]), which interrupts the substance transfer through the bacteria cell surface and thus affects their metabolism and inhibits bacterial growth. In addition, the positively charged property of chitosan could also achieve hemostatic functions because the electrospun chitosan network is able to gather up the negatively charged red blood cells and platelets to coagulate and stop bleeding [18]. Together with the affinity to mucous membranes and swelling capability [64], these advantages enable chitosan to be a commendable food polymer for the delivery of phytochemicals and drugs. Considering its safety aspects, chitosan belongs to a dietary supplement that has been regulated by the Dietary Supplement Health and Education Act (DSHEA) in the U.S. The FDA has also approved chitosan and related materials to be applied in some wound dressing bandages and hemostatic products [65,66].

Electrospinning chitosan alone was relatively difficult. Chitosan dissolves readily in aqueous solutions of organic acids such as formic/acidic acid. However, the high viscosity of chitosan acidic solution hindered the removal of solvents and the formation of nanofibers during electrospinning. Moreover, the charged nature of chitosan generates a repulsive force within the polymer molecules when interacting with the high-voltage electric field during electrospinning, yielding products such as large beads, aggregates, and discontinuous fibers instead of smooth nanofibers [67,68]. Harsh solvents such as concentrated acetic acid (AA) and trifluoroacetic acid (TFA) were able to overcome the above issues and satisfying electrospun nanofibers were produced [69,70]; however, solvents might introduce unwanted chemical reactions to chitosan and, therefore, be unsuitable for manufacturing. In order to properly electrospin chitosan, researchers tend to blend it with other biodegradable polymers. Polyethylene oxide (PEO) and polyvinyl alcohol (PVA) are popular choices for blending due to their well-recognized safety as food/drug additives, solubility in water, and compatibility with chitosan [71]. Other slowly biodegradable polymers such polycaprolactone (PCL), polylactic acid (PLA), and more could also be blended with chitosan for external use, with TFA, dichloromethane (DCM), or hexafluoro-isopropanol (HFIP) in their solvent systems [72,73].

**Figure 4:**
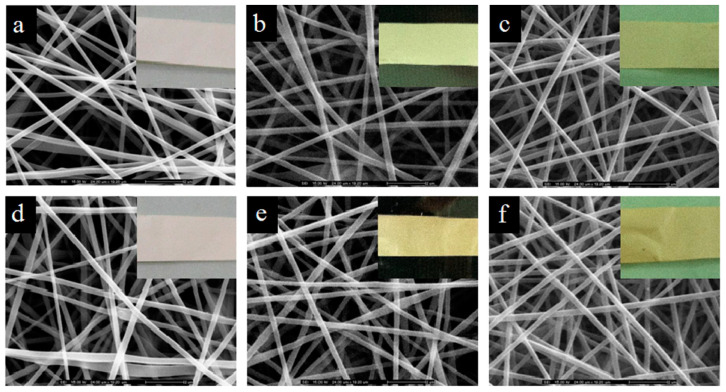
General appearances and SEM images of electrospun chitosan/PVA nanofiber films loaded with α-mangostin and stored at 25 °C with 40% relative humidity (RH) (**a**–**c**) or 45 °C with 75% RH (**d**–**f**) for 0, 3, and 6 months, respectively. Reused with copyright permission from [64].

Process details in typical cases of chitosan-blend electrospinning are as follows. Hosseini et al. fabricated an electrospun chitosan/PVA membrane for active packaging use. For the polymer blend, 1% chitosan solution (in 1% AA) and 12% PVA aqueous solution were mixed at a 2:8 ratio, and fish-based antioxidative peptides were incorporated as active components. During electrospinning, the voltage was applied at 15 kV over 15 cm with a feeding rate of 0.2 mL/h [74]. Charernsriwilaiwat et al. prepared electrospun chitosan–ethylenediaminetetraacetic acid (EDTA)/PVA nanofiber films loaded with α-mangostin extracted from garcinia mangostana (general appearance and SEM images are shown in Figure 4). The polymer blend was prepared by mixing 2% (*w*/*v*) chitosan–EDTA (2:1) solution and 10% (*w*/*v*) PVA solution at the ratio of 3:7. Then, α-mangostin (1–3 wt% of the polymer) was added to the blend. The spinning electric field was 15 kV while the solution was fed at 0.25 mL/h using a 20 G needle (diameter = 0.9 mm) [64]. Elhamalsadat et al. fabricated electrospun chitosan/xanthan nanofibers loaded with curcumin. In their experiment, 3% (*w*/*v*) of chitosan, 0.75% (*w*/*v*) of xanthan, and 2% (*w*/*v*) of curcumin were dissolved in formic acid to form the polymer blend and successfully form continuous fibers with some small nodes. The electrospun condition was set at 25 kV over 10 cm while the polymer blend was fed at 0.6 mL/h using a 21 G needle [75]. Zhou et al. developed an electrospun chitosan oligosaccharide (COS) and PCL composite nanofiber loaded with rutin and quercetin. Amounts of 20% (*w*/*v*) PCL and 2–4% (*w*/*v*) COS with 1% (*w*/*v*) rutin or quercetin were mixed in a solvent system comprised of formic and acetic acid (7:3). During electrospinning, the electric field was set at 32 kV (29 kV at needle tip, −3 kV at collector) over 15 cm. The feeding rate was 0.77 mL/h using a 21 G needle [76].

The bioactive compound release behavior of electrospun chitosan nanofibrous composites tend to be a rapid process due to its high affinity to aqueous environments and swelling ability as previously mentioned. From the cumulative dissolution data of the electrospun chitosan/PVA membrane fabricated by Hosseini et al., the loaded active peptides release logistically for around 45 min to reach equilibrium while the release efficiency was inversely related to the loading amount (~45% release for 1 mg/mL peptides versus ~25% release for 5 mg/mL peptides) [74]. The release profile of α-mangostin from this chitosan/PVA composite fibrous mat prepared by Charernsriwilaiwat et al. was a typical burst release. Approximately 80% α-mangostin was released within 30 min for all the samples and the equilibrium was reached at around 2 h [64]. Curcumin release at physiological pH from the chitosan/xanthan nanofibers by Elhamalsadat et al. was an extended profile and took around 2 h to reach the equilibrium. The final release amount of curcumin stayed at about 45% of the total loading [75]. Dissolution data from the electrospun COS and PCL composite quercetin/rutin nanofiber by Zhou et al. revealed a typical burst release profile of rutin (less than 2 h) and quercetin (around 10 h) [76].

Nevertheless, some researchers were able to achieve the controlled release of bioactive compounds from chitosan composite nanofibers through encapsulation. Fahimirad et al. fabricated electrospun chitosan and PCL-blended nanofibers loaded with curcumin–chitosan nanoparticles. The nanoencapsulation strategy successfully achieved the controlled release of curcumin to more than 80% of its total loading for 15 days [65]. Zou et al. prepared electrospun chitosan/PCL nanofibers loaded with chlorogenic acid with halloysite nanotube encapsulation. The nanotube encapsulation prolonged the release of chlorogenic acid to at least 10 days; if the release study could be extended to 1 month, it is expected that more release might be observed [77]. Coaxial electrospinning was another technique to achieve controlled release profiles for electrospun chitosan. Poornima et al. developed a core–shell electrospun nanofiber with low Mw chitosan as the core and PCL as the shell. The core chitosan solution was loaded with resveratrol and ferulic acid. The release results indicated a logistic-controlled release profile for 5 days [78]. Brief comparisons with other polysaccharides are listed in Table 1. Electrospun formulations and release profiles are summarized in Table 2.

### 2.4. Alginate

Alginate is another charged food polymer (anionic) that has been broadly studied. It is abundantly found in brown algae and consists of glycosidic 1 → 4 linked β-D-mannuronic acid and α-L-guluronic acid. However, the arrangements of these two units are influenced by algae types, seasons, and other environmental conditions [79]. Sodium alginate is able to gel with Ca^2+^ and a series of ions, which indicates it could form gels by interacting with Ca^2+^ from the bleeding wound spots to maintain a moist local condition and reduce harmful bacterial contact, while providing drug delivery at the same time. These advantages enable sodium alginate to be a popular choice for wound dressing. Considering it safety aspects, sodium alginate is approved by the U.S. FDA and regulated under the Code of Federal Regulations Title 21. It is also approved to apply in some wound treatment medical products and dental materials [80,81,82]. Moreover, sodium alginate does not interact with cells apparently, and thus it has satisfying cytocompatibility and low concern in allergic reactions [81,83].

**Figure 5:**
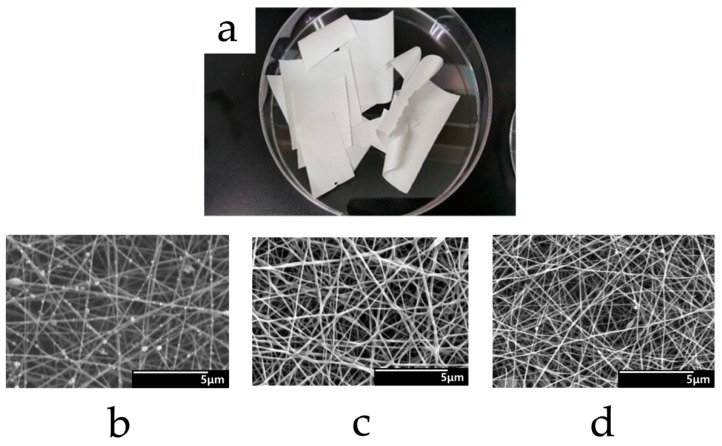
General appearance (**a**) and SEM images (**b**–**d**) of electrospun alginate/CMC/PEO lidocaine nanofiber films with total polymer concentration at 5% (*w*/*v*), 7% (*w*/*v*), and 9% (*w*/*v*). Reused from [85] under the terms and conditions of the Creative Commons Attribution (CC BY) license (http://creativecommons.org/licenses/by/4.0/ (accessed on 24 March 2023)).

Electrospinning pure alginate into a defect-free nanofibrous product has been challenging due to its low Mw, gel-forming, and charged properties that affect the surface tension of its solution [81,84]. This situation is somewhat similar to chitosan, and the alginate solution is also similar to chitosan in its ability to blend with other biocompatible hydrophilic polymers such as PEO and PVA, which are more compatible with alginate and less complicated in solvent system design. Baek et al. developed an electrospun lidocaine-loaded anti-adhesion nanofiber film with alginate, carboxymethyl cellulose (CMC), and PEO at a weight ratio of 1:1:0.22 with just water as the solvent. The total polymer concentration was 5% (*w*/*v*), 7% (*w*/*v*), and 9% (*w*/*v*). The electrospun condition was 25 kV over 10 cm while the feeding rate was 0.5 mL/h using a 25 G needle tip. Referring to the SEM pictures shown in Figure 5, continuous defect-free fibers were yielded except for the 5% polymer sample, which showed the thinnest fibers with the formation of some random beads.; this might be due to the critical concentration not being reached with inadequate viscosity/polymer molecule entanglement [85]. Gonzalez et al. studied the electrospun fibers made with varied ratios of low (600 kDa, from 0.75% to 0.05% *w*/*w* in water) and high Mw (4000 kDa, from 1.30% to 2.07% *w*/*w* in water) PEO mixing with 1% sodium alginate. During electrospinning, varied conditions were applied including 15 cm/15 kV, 20 cm/20 kV, 20 cm/25 kV, and 25 cm/25 kV while feeding at 0.8 mL/h. The results indicated that at a specific ratio range of high/low Mw PEO, which was 0.3–1, the fiber mechanical properties were significantly enhanced without a change in fiber size due to the increase in chain entanglement between the PEO and alginate [86]. It could be inferred that, for electrospinning alginate/PVA blends, the more viscous the chosen alginate, the higher the ratio of alginate that can be blended with PVA. In addition, blending alginate with hydrophobic biocompatible polymers for electrospinning was actually feasible by applying special techniques such as dual-jet “knitting”, emulsion electrospinning, and more. Hu et al. fabricated an alginate/PCL composite fiber mat by dual-jet co-electrospinning 2 strands of polymer nanofibers at the same time, under different electric fields (20 kV for alginate and 10 kV for PCL) with varied feeding rates (alginate: 0.25 mL/h, PCL: 0.16 mL/h). This technique avoided relying on harsh solvent systems. The final product could provide both a moist isolation environment by alginate and better cell attachment by PCL [87]. Xu et al. prepared an alginate/PLA nanofiber film by emulsion electrospinning technique. In detail, the emulsion blend was made by adding 0–5 mL of alginate solution (40 mg/mL aqueous) totally to 18 mL of chloroform with 0.3 g Span80, and 1.92 g PLA was incorporated subsequently and gradually. High-speed stirring at 7000 rpm was required to generate the emulsion before electrospinning. The electric field was 15 kV over 15 cm with an emulsion feeding rate of 0.5 mL/h. From their SEM graphs, all the formulations were successful in yielding continuous and evenly distributed fibers. The addition of alginate significantly decreased the fiber diameter, from ~800 nm (pure PLA) to ~200 nm (emulsified alginate + PLA), which largely enhanced the surface area of the film. Increasing the amount of alginate addition slightly decreased the fiber diameter [88].

Release studies of electrospun alginate blend fibers usually demonstrated fast release profiles. Najafi et al. prepared an electrospun alginate/PVA blend nanofiber film loaded with 5–15 wt% of cardamom extract (CE). The release of CE was more rapid with the increase in CE loading in the nanofibers. The film with 5% CE reached release equilibrium at around 2 days while the one with 10% CE burst-released for about 20 h to reach the maximum concentration [89]. Dede et al. fabricated an electrospun film using a natural zein/alginate emulsion blend loaded with 1–20% *v/v* of basil oil. From their cumulative release curve, the release of estragole (one of the typical components of basil oil) kept increasing throughout their 2 h examination, longer release duration should be considered to observe the equilibrium timing. In addition, the release percentage increased together with the increase in bioactive compound loading amount. At 2 h of experiment, 1% loading film released about 30% of estragole while 20% loading film released around 70% [90]. Rezaei et al. studied an electrospun alginate/sodium CMC film loaded with curcumin. From the dissolution data, curcumin was burst-released from the alginate/CMC film within 2 h and then gradually reached equilibrium in the next 10 h. The film with 3% curcumin loading actually had a higher release percentage (~30%) compared with 5% curcumin loading film (~20% release) [91]. Brief comparisons with other polysaccharides are listed in Table 1. Electrospun formulations and release profiles are summarized in Table 2.

### 2.5. Hyaluronic Acid

Hyaluronic acid (HA) is a natural linear glycosaminoglycan comprised of heterogeneously arranged D-glucuronic and N-acetyl-D-glucosamine residues. Polysaccharide HA has a wide range of Mw, in which the low Mw HA (1–25 × 10^4^ Da) and high Mw HA (>1 × 10^6^ Da) were frequently studied since they were actively involved in the cell signaling pathways during wound healing [92]. HA is readily found in the connective tissues and extracellular matrix (ECM) in humans and is well recognized as a biocompatible, highly biodegradable hydrophilic polysaccharide for wound healing and many other regenerative biomedical applications, with the functions of modulating cytokines production, inflammatory responses, collagen generation/fibroblast proliferation, and scar repairing [92,93,94]. Considering its safety aspects, sodium HA with an Mw range of 10–4000 kDa produced by microbial fermentation is FDA approved in GRAS Notice No. 976, in accordance with Section 201(s) of the Federal Food Drug and Cosmetics Act [95,96].

**Figure 6:**
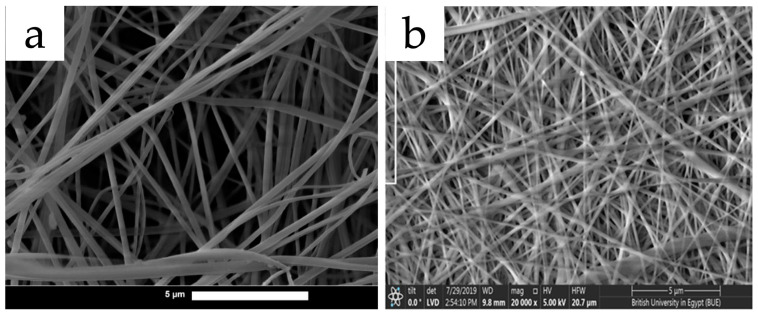
SEM images of electrospun active-compound-loaded nanofibers based on pure HA (**a**) and PVA-blended HA (**b**). Electrospun pure HA tends to generate more randomly distributed fibers with some adhering pieces; electrospun-blended HA fibers can be more uniform and intact. Reused with copyright permission from [99] and from [97] under the terms and conditions of the Creative Commons Attribution (CC BY) license (http://creativecommons.org/licenses/by/4.0/ (accessed on 4 May 2023)).

HA is water soluble and can form viscous aqueous solutions, however, it is hard to electrospin HA aqueous solutions due to extensive H-bonding and electrostatic interactions occurring in between the HA molecules in water. These interactions cause the HA polymer chains to be rigid to the extent that they cannot generate enough chain entanglements for electrospinning. To electrospin pure HA, solvent systems with high pH or organic solvents such as DMF/DMSO need to be utilized to reduce the surface tension [93]. In recent years, few studies on electrospun pure HA have been reported. Snetkov et al. reported an electrospun pure HA membrane loaded with curcumin. They utilized a solvent system containing an equal amount of water and DMSO to dissolve 1.9 wt% of HA. In the electrospinning process, humidity was controlled at 30–35%, while the electric field was set at 28 kV at a distance of 15 cm, and the feeding rate was 2 mL/h. From the microscopic graphs, the pure HA fibers were generally intact with a relatively wide distribution in diameter and thick/adhering pieces of fibers were observed (demonstrated in Figure 6a) [97]. Nevertheless, most of the researchers tended to co-electrospin HA with other biodegradable polymers, which were more cost effective with fewer concerns compared with toxic solvents. Janmohammadi et al. fabricated a PCL/HA blend electrospun scaffold loaded with L-ascorbic acid. In their formulation, the solvent was formic acid/acetic acid (7:3). Total concentration of PCL and HA was 8% with weight ratios of 9:1, 8.5:1.5, and 8:2. The electric field was set at 15 kV with a 10 cm distance and the feeding rate was 0.1 mL/h. SEM images displayed that massive and highly intercrossing webs of nanofibers were formed without beads, whose diameter was less than 200 nm [98]. Hussein et al. prepared electrospun PVA/HA nanofibers loaded with both L-arginine and citric acid. The solvent for the polymer blend was pure water and the concentrations were 10% *w/v* for PVA and 2% *w/v* for HA solutions mixed at 8:2, 6:4, and 5:5. The electric field was set at 30 kV in 15 cm, with a feed rate of 0.3 mL/h. SEM data reflected that dense, uniform, and defect-free nanofibers were yielded (demonstrated in Figure 6b) [99]. Xu et al. fabricated an electrospun fibrous facial mask with PVA and HA loading crude huangshui polysaccharide. The solvent system was 90 °C hot water dissolving 8% PVA and 0.2 g HA (*w*/*w*). The electric field was 22 kV allocated at 15 cm while the polymer blend was fed at 1 mL/h. The electrospun fibers appeared intact and highly uniform with a diameter of around 200 nm by SEM [100].

Dissolution studies against pure HA or HA-blend electrospun fibers demonstrated varied release profiles. Curcumin-loaded electrospun pure HA nanofibers by Snetkov et al. demonstrated controlled release profile for two hours with a complete release of loaded curcumin at the end of dissolution [97]. An L-ascorbic-acid-loaded PCL/HA blend electrospun scaffold by Janmohammadi et al. was reported to have a controlled release profile for 14 days. The best result was from the PCL/HA (8:2) fiber scaffold which had a final release of 70% encapsulated ascorbic acid [98]. L-arginine-loaded electrospun PVA/HA nanofibers prepared by Hussein et al. demonstrated a generally controlled release profile for 2 days with a maximum release of around 90% of the loaded compound [99]. The huangshui-polysaccharide-loaded electrospun PVA/HA nanofibrous facial-mask fabricated by Xu et al. was reported to burst release within 15 min to about 50% of its loaded compound [100]. It could be inferred that the hydrophilicity of the loaded bioactive compound was not always the decisive factor of release profiles. Polymer blends with high polarity, such as PVA/HA or pure HA, tend to provide a release process of less than two days to even hours. The combination of a relatively low-polarity polymer with HA, such as PCL/HA, could offer an appealing controlled release profile for a few weeks. Brief comparisons with other polysaccharides are listed in Table 1. Electrospun formulations and release profiles are summarized in Table 2.

**Table 1:** Brief comparison among popular food polysaccharides for electrospinning.

Polysaccharides	Advantages	Disadvantages
Starch	High acceptance, safety, and availability.	Mechanically weak; difficult to electrospin
Cyclodextrin	Hollow truncated structure to form inclusion complex with bioactive compounds.	Needs blending with other biodegradable polymers for controlled release.
Chitosan	Positively charged properties natively against microbials; high affinity to mucous membranes; and swelling properties.	Needs blending with other biodegradable polymers to electrospin intact fibers; often requires an acidic solvent system.
Alginate	Able to gel with Ca^2+^ for hemostasis applications; maintains wound moisture.	Needs blending with other biodegradable polymers to electrospin intact fibers; short release durations.
Hyaluronic acid	Able to gel at low concentration; highly biocompatible and biodegradable; and modulates cell signaling during scar repair.	Often requires blending with other biodegradable polymers to electrospin.

**Table 2:** Popular electrospun food polysaccharides and related release profiles.

Polysaccharides	Polymer Blend and Solvent Systems	Bioactive Compounds	Release Profiles
Starch	80% amylose corn starch in 95% DMSO, boiling bath [29].	Not loaded	N/A
	Common corn starch in 2% (*w*/*w*) NaOH solution [30].	Not loaded	N/A
	High amylose starch in 95% DMSO, boiling bath [36].	Palmitic acid	N/A
Cyclo-dextrin	HP-β-CD inclusion complex + PVA in water [46].	Berberine	Extended release to 4 h
	γ-CD inclusion complex + pullulan in water [55].	Eugenol	Burst release in less than 2 min
	HP-β-CD (polymer free) in water [56].	Cuminaldehyde	Burst release in less than 5 s
	HP-γ-CD, HP-β-CD inclusion complex (polymer free) in 60 °C hot water [57].	Curcumin	Burst release in less than 5 s
	HP-β-CD inclusion complex (polymer free) in water [48].	Gallic acid	Burst release in less than 5 s
Chitosan	Chitosan in 1% acetic acid + PVA in water [74].	Fish peptides	Burst release in 45 min
	Chitosan-EDTA/PVA in water [64].	α-mangostin	Burst release 80% in 30 min
	Chitosan/xanthan in formic acid [75].	Curcumin	Extended release in 2 h
	Chitosan oligosaccharide/PCL in formic + acetic acid [76].	Rutin, quercetin	Burst release in 2 h (rutin) and 10 h (quercetin)
	Nanoparticle: chitosan in 2% acetic acid. Polymer blend: chitosan/PCL in 7:3 DCM:DMF [65].	Curcumin	Controlled release for 15 days
	Nanotube: halloysite in 60% ethanol [77]. Polymer blend: chitosan/PCL in 90% acetic acid.	Chlorogenic acid	Controlled release for 15 days
	Shell: PCL in DCM. Core: chitosan in 90% acetic acid [78].	Resveratrol and ferulic acid	Controlled release for 5 days
Alginate	Sodium alginate/PVA in water [99].	Cardamom extract	Extended release in 20 h
	Zein in ethanol + alginate in water (emulsion) [90].	Basil oil	Burst release 70% in 2 h
	Alginate/CMC Na in DMSO/DMF [91].	Curcumin	Burst release 30% in 2 h, equilibrium at 10 h
	Pure HA in water/DMSO [97].	Curcumin	Controlled release for 2 h
	HA/PCL in 7:3 formic acid/acetic acid [98].	Ascorbic acid	Controlled release for 14 days
Hyaluronic acid	HA/PVA in water [99].	L-arginine	Controlled release for 2 days
	HA/PVA in water [100].	Huangshui polysaccharide	Burst release in 15 min

## 3. Diverse Applications of Electrospun Food Polysaccharides

In addition to the bioactive compound release functions discussed in the last section, electrospun food polysaccharides were also capable of achieving a series of physical, chemical, and biomedical functions that facilitate our daily lives. In this section, several frequently researched functions of electrospun food polysaccharides are elaborated. Summaries of the popular applications are listed in Table 3.

### 3.1. Active Food Packaging

**Figure 7:**
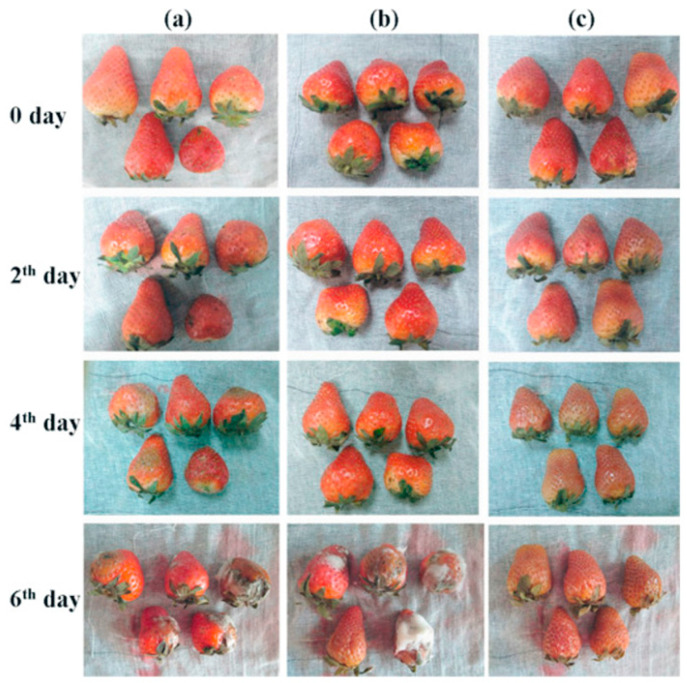
Strawberries stored at 21 °C and wrapped with (**a**) no wrapping (control); (**b**) commercial food wrap; and (**c**) electrospun PVA/cinnamon oil/β-CD nanofibers. Reused with copyright permission from [103].

For applications in food packaging, electrospun polysaccharides are frequently utilized in multiple ways. They can serve as reservoirs to load anti-bacterial essential oils to provide a long-term anti-bacterial effect, or to load antioxidants as oxygen scavenging packaging. Lu et al. developed an oregano essential oil alginate/PEO film by electrospinning. The polymer formulation was prepared by mixing 3.75 wt% alginate and 4 wt% PEO aqueous solutions at 4:1 blended with oregano oil, which was also crosslinked by CaCl_2_ to enhance the mechanical properties for packaging use. The oregano oil nanofibers were demonstrated to inhibit the growth of several food-borne pathogens including *S. aureus*, *P. aeruginosa*, *S. Typhimurium*, and *Listeria monocytogenes* [101]. Shi et al. also utilized oregano essential oil to develop active food packaging. They electrospun PLA and PCL with more hydrophobicity and heat stability; it was loaded with oregano oil-β-CD complex to achieve longer release (from 6 to 10 days at 4 °C or from 4 to 6 days at 25 °C) and an antimicrobial/antifungal effect [102]. Wen et al. fabricated electrospun cinnamon oil/β-CD/PVA/antimicrobial nanofibers using water as solvent system with constant stirring and heating. The results showed that the β-CD encapsulation and PVA matrix largely enhanced the heat stability of cinnamon oil, from less than 100 °C to almost 300 °C. Minimum inhibitory concentration (MIC) results indicated that the antimicrobial effect of electrospun PVA/cinnamon oil/β-CD at 8.9–9.9 μg/mL was as effective as 4.0–5.0 μg/mL kanamycin sulfate against *S. aureus* and *E. coli*. They also conducted a practical analysis to demonstrate that the electrospun cinnamon oil film kept strawberries intact for 6 days at 21 °C compared with the control which was apparently moldy (demonstrated in Figure 7) [103]. The charged property of chitosan also makes it a good choice for an antimicrobial packaging material. Arkoun et al. prepared an electrospun chitosan/PEO composite nanofiber film. The solvent system was 50% (*v*/*v*) acetic acid dissolving 3–7% (wt/v) of chitosan with a chitosan/PEO ratio of 4:1. It was found that 1 cm^2^ of the chitosan/PEO film was highly effective in reducing *S. typhi* (~2 logs), *E. coli* (~4 logs), *L. innocua* (~4 logs), and *S. aureus* (~4 logs) [104]. Similar antibacterial effects were also proven in other electrospun chitosan blend nanofiber membranes [105].

For the oxygen-scavenging type of packaging, Fonseca et al. successfully fabricated electrospun potato starch nanofibers loaded with carvacrol. Although modifications of starch often compromised edibility, they are useful in food packaging because of the well-enhanced physical properties. The solvent system was 75% formic acid which led to the acetylation of starch to become starch formate [106]. From the characterization results, their highest loading product suppressed 83.1% of oxidative activity from ABTS radicals (2,2’-azino-bis 3-ethylbenzothiazoline-6-sulfonic acid). The electrospun films also enhanced the thermal stability of the loaded carvacrol from around 160 °C to 200–250 °C [107]. Duan et al. fabricated an active packaging nanofiber utilizing chitosan/gelatin and loaded it with 0.1%, 0.2%, and 0.3 wt% curcumin to achieve oxygen scavenging effects. Results of 1,1-diphenyl-2-picrylhydrazyl (DPPH) antioxidation assay indicated about 25.5%, 41.3%, and 51.2% oxidative radical scavenging activities [108]. These properties including anti-oxidation, anti-microbial, and enhanced heat-tolerance were valuable attributes of packaging to protect food from the impact of environmental oxygen, heat, and microbiological contaminations.

In addition, electrospun polysaccharides with bioactive compounds could also become a pH-indicator type of active packaging by utilizing the pH-color dependency of certain compounds. Yildiz et al. prepared a chitosan/PEO nanofiber loaded with curcumin to monitor chicken freshness. Their chitosan/PEO (2.5/7.5 by weight) fibers demonstrated apparent sensitivity to the change in surface pH and total volatile basic nitrogen (TVB-N) over time on the chicken surface, from bright yellowish to darker yellowish/red in color [109]. Shavisi et al. electrospun a chitosan–gum Arabic nanofiber loaded with anthocyanins that are famous for their pH-color correlations. The encapsulated *Rosa damascena* anthocyanin was pink to red at acidic pH while dark yellow to brown at neutral to basic pH, which were good indicators for acid and protein rancidity of chicken meat [110].

### 3.2. Filtering against Particles, Volatiles, Heavy-Metal Ions, and Microorganisms

Electrospun nanofibrous films are well recognized for their high surface area and porous structure. They are also highly tunable in chemical activities and mechanical properties, thus allowing them to become a preferred choice for filtering materials. Cyclodextrin has a hydrophilic surface and thus is unable to filter water-based liquid; nevertheless, electrospun CD was able to achieve air-filtering functions against microparticles and volatile organic compounds (VOCs). Wang et al. electrospun PVA/β-CD composite nanofibers to achieve high-efficiency air filtering. In the polymer blend solution, 12% (*w*/*v*) was the concentration of PVA while the ratio of β-CD/PVA was 0.5:1, 0.75:1, 1:1, and 1.25:1, with water as the solvent system. After detection, their electrospun composite was able to remove over 95% of the particulate matter (PM) of 2.5 particles with the concentration of 1000 μg m^−3^ for as long as 30 h [111]. Wanwong et al. developed an electrospun PLA/CD membrane for both PM and VOC removal. In their formulation, PLA (10% *w*/*v*) was dissolved in a solvent system of DCM/DMF (dimethylformamide) at 7:5 (*v*/*v*), and the CD addition ratio was 2.5%, 5%, and 10% by weight of PLA. From the filtering results, the 2.5 wt% CD/PLA electrospun membrane achieved over 95% of PM2.5 and PM10 removal for 20 min. VOC removal represented by a toluene vapor entrapment test demonstrated that the 2.5 wt% CD/PLA sample removed 90% of toluene within 5 min [112].

**Figure 8:**
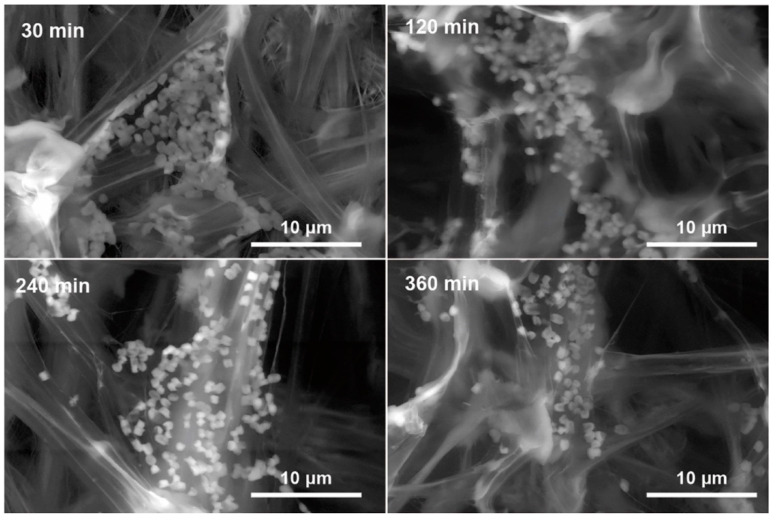
SEM images of *E. coli* bacteria being filtered on the positively charged nylon/chitosan nanofibers. Reused from [119] under CC BY-NC-ND license (http://creativecommons.org/licenses/by-nc-nd/4.0/ (accessed on 3 April 2023)).

Besides air particle filtration, the swelling, porosity, high surface area, and positively charged properties of electrospun chitosan enable it to be effective in entrapping heavy-metal ions [113,114,115] and air-borne bacteria [116,117,118]. Sun et al. prepared an electrospun nylon/chitosan nanofibrous filter. In the formulation, 15 wt% nylon-6 was mixed with chitosan at 4:1 in formic acid. The electrospun nylon/chitosan filter exhibited a more than one-log reduction in *E. coli* in the test airflow, and the bacteria filtered on the nanofibers are displayed in Figure 8 [119]. Li et al. electrospun chitosan-nanoparticle-encapsulated PLA fibrous membranes with antibacterial air-filtering properties. An amount of 2.5 wt% of chitosan nanoparticles+ and 8 wt% PLA were dissolved in DCM/DMAc (dimethylacetamide) at 10:1. A more than two-log reduction for both *S. aureus* and *E. coli* was achieved with this membrane. Furthermore, it also has excellent air particle filter ability that decreased PM2.5 from 999 μg/m^3^ to 0 μg/m^3^ in about half an hour in a space of 0.125 m^3^ [73]. Lee et al. developed electrospun PLA/chitosan mats with a polymer blend consisting of ~30 wt% PLA and ~6 wt% chitosan in the solvent system of pure TFA. It was reported to absorb up to 1 mg silver ion/g chitosan (in the form of electrospun nanofibers) [120]. The gel-forming property of alginate has also been utilized for heavy metal removal. Wang et al. constructed a novel poly (acrylic acid)–alginate nanofibrous hydrogel by electrospinning. This electrospun nanofiber gel had excellent mechanical properties of ~16 MPa tensile strength and ~200% elongation. It exhibited a prominent Cu(II) removal capability of close to 600 mg/g and satisfying reusability after recycling 7 times [121].

### 3.3. Catalytic Functions/Enzyme Immobilization

Catalysts enhance the speed of chemical reactions and could theoretically be recycled after reactions. Some catalysts, such as platinum-containing compounds or bioengineered enzymes, are so costly that their reusability and immobilization has become a popular research area. Fortunately, electrospun nanofibrous membranes have native advantages such as high porosity and surface–volume ratio, which make it an attractive candidate for loading bioactive enzymes as an immobilization tool.

**Figure 9:**
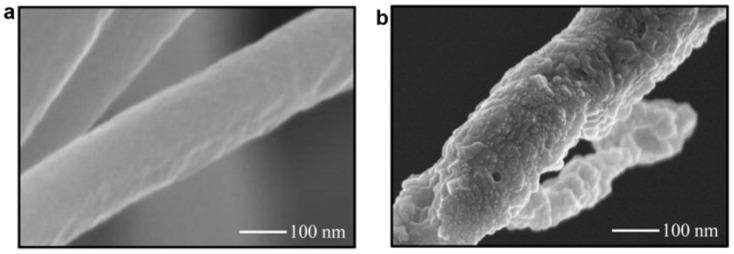
(**a**) Chitosan/PVA nanofibers. (**b**) Chitosan/PVA nanofibers after GA crosslinking and lysozyme immobilization. Reused with copyright permission from [123].

Işik et al. immobilized arginase in electrospun PVA/β-CD nanofibers with Mn^2+^ ion (to enhance arginase activity) which was crosslinked by glutaraldehyde (GA). An amount of 6 wt% PVA, 3 wt% β-CD, and 1 U/mL arginase (44.45 U/mg protein) was tested to be the optimal formulation. Thermal stability assay indicated that their PVA/β-CD/arginase nanofibers maintained over 80% of arginase activity at a wide temperature range of 20–70 °C, while the free arginase maintained the same activity only at a narrow range of 35–50 °C. In addition, recycle assay indicated that their sample maintained ~50% of activity after 20 times of use and could be stored for 30 days maintaining ~75% of activity. In comparison, free arginase maintained only ~30% activity. Data indicated a successful immobilization of bioactive enzymes by electrospun β-CD-based nanofibers [122]. Park et al. electrospun a lysozyme-immobilized chitosan nanofiber for bacterial removal. A 3 wt% chitosan solution (in 2% acetic acid) and 9 wt% PVA were mixed at 1:3 as the polymer blend. The electrospun chitosan/PVA was then crosslinked with GA, washed, and treated with lysozyme solution (5 g/L). SEM images of nanofibers before and after lysozyme immobilization are shown in Figure 9b. The results demonstrated that the electrospun membrane immobilization enhanced the enzyme activity, especially at non-optimum temperature ranges. At 25–30 °C and 50–55 °C, the lysozyme in electrospun form could achieve up to about 20% more activity than the free form. At 60 °C, the electrospun product still maintained enzyme activity for more than 3 h while the free form lost its activity at 1.5 h. The reusability assay showed that after 100 re-uses the lysozyme activity still remained [123]. Kamaci et al. developed a phytase-immobilized electrospun PVA/alginate nanofibrous membrane. They mixed sodium alginate (2%, *w*/*v*) and PVA (10%, *w*/*v*) in 0.1 M sodium acetate (pH 5.0) buffer solution at a 4:1 ratio, and subsequently added their self-extracted phytase (0.5– 2.0% *v*/*v*) to form their polymer blend for electrospinning. From their stability assays, the electrospun nanofibers maintained more than 70% of enzyme activity at a wide range of pH 2–9, while the free enzyme lost its activity significantly at pH 6–9. For the heat tolerance study, the electrospun form maintained over 70% of enzyme activity at 80 °C for 30 min; however, the free enzyme lost activity after 10 min [124]. It could be inferred that food polysaccharides had satisfying performances in enzyme immobilization that enhanced heat tolerance, effective pH range, reusability, and more.

### 3.4. Wound Dressing

**Figure 10:**
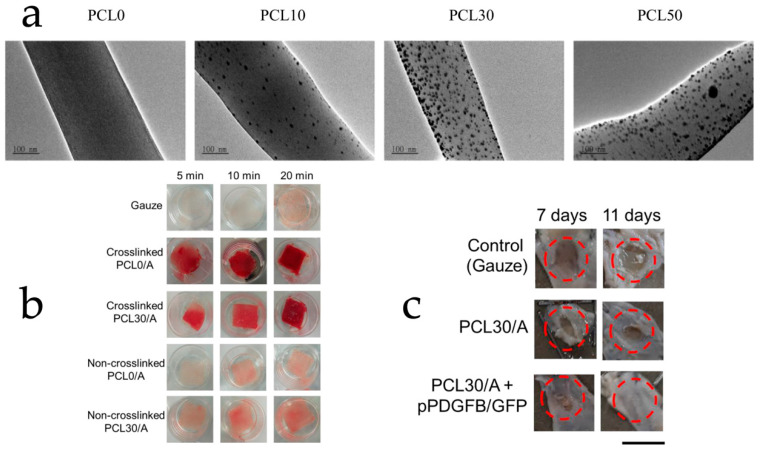
(**a**) SEM images of alginate/PCL nanofibers loading 0, 10, 30, 50 mM silver ions. (**b**) Blood clotting experiment showing the hemostasis efficacy of Ca^2+^ crosslinked alginate/PCL nanofibers. (**c**) Wound healing experiment displaying faster closure by alginate/PCL nanofibers. Reused with copyright permission from [87].

For wound dressing materials, the ability of hemostasis, isolation, and anti-infection are crucial. Electrospun polysaccharides loaded with bioactive compounds are capable of providing high affinity to wound tissue, bacteria removal, gelling isolation, and more, which indicates their potential in the wound dressing function. Modified starch has been utilized in some wound dressing studies for its enhanced mechanical properties and the ease of crosslinking. Adeli et al. constructed an electrospun PVA/chitosan/starch membrane. For the polymer blend, PVA solution 9% (*w*/*v*) in water and 2% (*w*/*v*) chitosan solution in acetic acid were mixed at 9:1 or 5:5 combining 0–15% (*w*/*v*) of starch in the final solution. After electrospinning, the membrane was crosslinked with GA. Antibacterial assay indicated that the higher the content of chitosan in the membrane, the more it could inhibit *S. aureus* and *E. coli*. From the scratch assay, the best wound closure efficiency was found for the membrane with a PVA/chitosan/starch ratio of 9:1:1, which means the electrospun membrane formulation could be tailored according to the wound types and situations [125]. Yang et al. developed an electrospun oxidized-starch nanofiber membrane crosslinked with antimicrobial ε-poly-lysine. A mechanical study revealed that this membrane has a tensile strength of 0.34 MPa and an elongation of 62.0%, which is more than twice that of a commercial alginate-Ag+ product. The water absorption assay, which was closely related to hemostasis performance, demonstrated an absorption result of 12.5 g water/g membrane that was close to the commercial one. Antimicrobial assay displayed a more than one-log reduction in *E. coli* for the membrane. An inhibitory zone against *S. aureus* was also observed, but not as significant as towards *E. coli* [126]. Cyclodextrin was frequently adopted to form inclusion complexes with anti-microbial bioactive compounds such as curcumin [127], citral [128], thymol [129], and more to assist wound healing. Nevertheless, Balakrishnan et al. developed an interesting electrospun membrane that utilized PVA/β-CD to reduce the added silver nitrate into silver nanoparticles to form a composite material for wound healing. The membrane was then decorated with riboflavin to enhance efficacies. Antibacterial assay indicated the effectiveness of the membrane against *S. aureus* and *E. coli*. The in vivo wound healing experiment proved that their electrospun membrane took 10 days to complete wound healing on lab mice while the control group wound only recovered less than 60% in the same time [130]. Chitosan, due to its positively charged nature against bacteria and swelling properties, is a popular choice for wound dressing development. Liu et al. fabricated a coaxial electrospun nanofiber matrix with two layers: PCL/chitosan and zein–curcumin. The solvent system contained acetic acid, chloroform, methanol, and water. From their burn wound healing study, the electrospun-matrix-treated group had the shortest recovery time (~23 days) compared with 2 commercial products “gauze wound dressing” (~35 days, GWD) and “silver ion alginate dressing” (~29 days, SIAD). Bacteria detection data showed that after 12 days of treatment, the GWD group decreased the total bacterial infection rate by ~14%, the SIAD group decreased by ~55%, and the electrospun matrix group decreased by ~78%. The scar repair study also found that the electrospun matrix had the best scar repair rate of ~59% compared with GWD at ~24% and SIAD at ~44%. The pain score (visual analog scale, VAS) and patient satisfaction score (self-rating anxiety scale, SAS/self-rating depression scale, SDS) also suggested that the electrospun chitosan/curcumin matrix was the most satisfying and alleviating treatment among the experimental groups [131]. Abdelbasset et al. developed a chitosan/CMC electrospun wound dressing loaded with 0.3% *w*/*w* mequinol. At day 7 and 14 of the in vivo wound healing study, this dressing showed ~60% and 96% of wound closure, which was about twice better than the control (sterile gauze). Histopathological examinations demonstrated that the electrospun dressing achieved ~56 µm epithelial thickness and ~38% collagen deposition that was much higher than the control with ~12 µm epithelial thickness and ~7% collagen deposition [132]. Hu et al. fabricated alginate/PCL composite electrospun nanofibers loaded with silver nanoparticles for wound dressing. The nanofibers were crosslinked by CaCl_2_ to enhance gelling properties. After crosslinking, the fibers reached a coagulation ratio of over 70% within 10 min compared with the control with only 40% (gauze), which proved its hemostasis capability. From the antimicrobial assays, over 70% removal against *S. epidermidis* and 1-log reduction in *E. coli* was achieved. From the in vivo wound closure study, the control group (gauze) mice only reached less than 60% wound closure at day 11. Nevertheless, the electrospun nanofibers achieved wound healing of 77% on day 7 and 95% on day 11. Details are provided in Figure 10 [87]. These evidences adequately demonstrate the potentials of electrospun polysaccharides loaded with bioactive compounds for wound healing purposes.

**Table 3:** Summary of several popular applications of food polysaccharides with/without bioactive compounds.

Applications	Polysaccharides	Bioactive Compounds	Functional Details
Active food packaging	Alginate/PEO	Oregano oil	Inhibited food-borne pathogens including *S. aureus*, *P. aeruginosa, S. Typhimurium,* and *Listeria monocytogenes* [101].
	β-CD complex + PLA/PCL	Oregano oil	Enhanced hydrophobicity and heat stability; controlled release for 10 days [102].
	β-CD complex + PVA	Cinnamon oil	Inhibited *S. aureus* and *E. coli*; extended strawberry shelf life; and enhanced the heat stability of cinnamon oil from ~100 °C to ~300 °C [103].
	Chitosan/PEO	Not loaded	Inhibited *S. typhi* (~2 logs), *E. coli* (~4 logs), *L. innocua* (~4 logs), and *S. aureus* (~4 logs) [104].
	Potato starch	Carvacrol	Suppressed 83.1% of oxidative activity from ABTS radicals; enhanced thermal stability of carvacrol from ~160 °C to ~250 °C [107].
	Chitosan/gelatin	Curcumin	Suppressed 51.2% of oxidative activity from DPPH radicals [108].
	Chitosan/PEO	Curcumin	pH and volatile nitrogen indicator for chicken freshness [109].
	Chitosan/gum Arabic	*Rosa damascena* anthocyanin	pH and volatile nitrogen indicator for chicken freshness [110].
Filtering	β-CD + PVA	Not loaded	A total of 95% of the PM2.5 removal for 30 h [111].
	CD + PLA	Not loaded	A total of 95% of PM2.5 and PM10 removal for 20 min; a total of 90% of VOC (toluene) removal within 5 min [112].
	Chitosan + nylon-6	Not loaded	A more than one-log reduction in *E. coli* in the test airflow [119].
	Chitosan nanoparticle + PLA	Not loaded	Two-log reduction in *S. aureus* and *E. coli*; ~100% removal of 999 μg/m^3^ PM 2.5 [73].
	Chitosan/PLA	Not loaded	Absorb up to 1 mg silver ion/g chitosan [120].
	Alginate/poly (acrylic acid)	Not loaded	A total of ~16 MPa tensile strength and ~200% elongation; Cu (II) removal ~600 mg/g after 7 recycles [121].
Enzyme immobilization	β-CD/PVA	Arginase	Enhanced enzyme thermal stability; >80% activity at 20–70 °C, while free arginase was active at 35–50 °C; maintained ~50% of activity after 20 recycles; and maintained ~75% of activity for 30 days, while free enzyme activity was ~30% [122].
	Chitosan/PVA	Lysozyme	Enhanced enzyme activity temperature range and thermal stability; activity remained after 20 recycles [123].
	Alginate/PVA	Phytase	Maintained >70% enzyme activity at a wide range of pH 2–9; enhanced thermal stability >70% activity at 80 °C for 30 min [124].
	Chitosan/starch/PVA	Not loaded	Inhibited *S. aureus* and *E. coli*; enhanced wound closure [125].
	Oxidized starch	Antimicrobial ε-poly-lysine	Tensile strength of 0.34 MPa and elongation of 62.0%, which was twice that of commercial alginate-Ag+ product; water absorption (hemostasis) close to commercial product; >1-log reduction in *E. coli*; and inhibition of *S. aureus* [126].
Wound dressing	β-CD/PVA	Silver nanoparticles	Enhanced wound healing with 10 days for wound closure on lab mice while the control only recovered <60%; inhibited *S. aureus* and *E. coli* [130].
	Chitosan/PCL + Zein	Curcumin	Reduced burn wound recovery time (~23 days) faster than commercial product (~35 days); bacterial inhibition rate (78%) > commercial (14%); scar repair rate (~59%) > commercial (24%); and pain/satisfaction score was higher than commercial products [131].
	Chitosan/CMC	Mequinol	Enhanced wound closure rate: 7 days ~60% and 14 days ~96% of wound closure, which was twice better than gauze; increased epithelial thickness and collagen deposition.
	Alginate/PCL	Silver nanoparticles	Enhanced blood coagulation (hemostasis) rate; removed 70% *S. epidermidis* and achieved 1-log reduction in *E. coli*; and enhanced wound closure: 95% closure on day 11, with 60% closure using gauze [87].

## 4. Conclusions and Future Perspectives

In summary, electrospun food polysaccharides are capable of achieving enhanced and/or controlled delivery of bioactive compounds while providing them with a shelter against environmental impacts. Advantages of popular electrospun food polysaccharides such as the inclusion complexing ability of the cyclodextrin family, natively charged/antibacterial properties of chitosan, and the gelling properties of alginate are highly useful in different fields. Polysaccharide electrospinning process parameters, polymer blend formulations, and the release characteristics of bioactive compounds elaborated in this review can assist researchers in the selection and matching of bioactive compounds/food polysaccharides and reduce the time required to review the literature. In addition to bioactive compound loading and releasing, other important functions of electrospun food polysaccharides such as active packaging, filtering, enzyme immobilizing, and wound healing were also discussed by reviewing case studies. This review elaborated the advantages and capabilities of food polysaccharides loaded with bioactive compounds and demonstrated their potentials in replacing some high-cost synthetic polymers while providing nearly the same efficacies and biocompatibilities.

Currently, there are still challenges in the utilization of electrospun polysaccharides with active compounds. For instance, modifications to starch and cellulose have brought great opportunities in expanding the choices of electrospinning materials; however, the recognition and approval issues of many modified starches and celluloses still remain. We hope more in vivo safety studies and clinical trials on modified polysaccharides will be carried out to push forward the utilization of modified polysaccharides, which could help to reduce the high cost of using synthetic biodegradable polymers such as PLA, PCL, etc. Solvent toxicity and residue removal is another issue in the fabrication of many electrospun polysaccharides. Techniques such as coaxial electrospinning is suggested to be promoted and researched more in order to reduce/avoid the use of toxic solvents for addressing the incompatibility between polymers and active compounds in electrospinning.
